# Preparation and Characterization of Semi-IPN Cryogels Based on Polyacrylamide and Poly(*N*,*N*-dimethylaminoethyl methacrylate); Functionalization of Carrier with Monochlorotriazinyl-β-cyclodextrin and Release Kinetics of Curcumin

**DOI:** 10.3390/molecules26226975

**Published:** 2021-11-18

**Authors:** Ecaterina Stela Dragan, Maria Valentina Dinu, Claudiu Augustin Ghiorghita, Maria Marinela Lazar, Florica Doroftei

**Affiliations:** Department of Functional Polymers, “Petru Poni” Institute of Macromolecular Chemistry, 700487 Iași, Romania; vdinu@icmpp.ro (M.V.D.); claudiu.ghiorghita@icmpp.ro (C.A.G.); mariperju@icmpp.ro (M.M.L.); florica.doroftei@icmpp.ro (F.D.)

**Keywords:** cryogel, poly(acrylamide), poly(*N*,*N*-dimethylaminoethylmethacrylate), monochlorotriazinyl-β-cyclodextrin, curcumin

## Abstract

Curcumin (CCM) is a natural hydrophobic polyphenol known for its numerous applications in the food industry as a colorant or jelly stabilizer, and in the pharmaceutical industry due to its anti-inflammatory, antibacterial, antioxidant, anti-cancer, and anti-Alzheimer properties. However, the large application of CCM is limited by its poor solubility in water and low stability. To enhance the bioavailability of CCM, and to protect it against the external degradation agents, a novel strategy, which consists in the preparation of semi-interpenetrating polymer networks, (s-IPNs) based on poly(*N*,*N*-dimethylaminoethyl methacrylate) entrapped in poly(acrylamide) networks, by a cryogelation technique, was developed in this work. All s-IPN cryogels were characterized by SEM, EDX, FTIR, and swelling at equilibrium as a function of pH. Functionalization of semi-IPN cryogel with monochlorotriazinyl-β-cyclodextrin (MCT-β-CD) led to IPN cryogel. The release profile of CCM from the composite cryogels was investigated at 37 °C, in pH 3. It was found that the cumulative release increased with the increase of the carrier hydrophobicity, as a result of increasing the cross-linking degree, the content and the molar mass of PDMAEMA. Fitting Higuchi, Korsmeyer–Peppas, and first order kinetic models on the CCM release profiles indicated the diffusion as the main driving force of drug release from the composite cryogels.

## 1. Introduction

Curcumin (CCM) is the main polyphenol found in the South Asian spice turmeric, (*Curcuma Longa*) responsible for the yellow colour. It is used as food additive, and due to its antioxidant, antimicrobial, anti-inflammatory, anti-cancer (prostate, colon, breast), nephro-protective, anti-aging, and potential anti-depressant properties, CCM has recently been one of the most investigated natural ingredients for biomedical applications [1,2,3,4]. However, the clinical application of CCM has some limitations, such as poor bioavailability, low water solubility and stability, and deficient cellular uptake determined by its hydrophobic structure. To enhance the therapeutic efficiency, numerous stimuli-responsive drug delivery systems (DDS) have recently been designed, the drug delivery being triggered by external or internal stimuli such as pH, temperature, light or magnetic field [2,5,6,7,8,9]. Among them, composites based on mesoropous silica, which allow the controlled release by gatekeepers, have recently been promoted [2,6,7,8]. Encapsulation of CCM into biocompatible polymers has been evaluated as a promising alternative to enhance its biomedical performances [10,11,12,13,14,15,16,17,18]. Thus, Dai et al. have found that zein–lecithin composite nanoparticles protected the loaded CCM against light and temperature much better than nanoparticles formed only with zein [10].

Hydrogels constitute another source for construction of various DDS [19,20,21]. Controlled delivery of CCM from synthetic polymeric membranes [22] and multifunctional composite films based on natural polymers [23] has recently been reported. Although many natural polymers can be used to produce hydrogels, the structural versatility characteristic to hydrogels based on synthetic polymers and their response to multiple external stimuli make them very attractive. Poly(acrylamide) (PAAm) is often used in the preparation of hydrogels for biomedical applications due to its affinity for proteins and other biomolecules. By their accessibility and biocompatibility, PAAm hydrogels constitute also one of the most investigated matrices in the preparation of semi-IPN composites, achieved by cross-linking polymerization of acrylamide in the presence of synthetic or natural polymers [24,25,26]. These hydrogels have numerous applications, such as DDSs [27,28], soil conditioner, and wastewater remediation [29]. Furthermore, the presence of amide groups constitutes an opportunity to get other structures by further reactions [30].

On the other hand, poly(*N*,*N*-dimethylaminoethyl methacrylate) (PDMAEMA) is a weak polycation well known for its antibacterial, hemostatic and anti-cancer properties [31] used in the preparation of new drug or gene delivery systems due to the good biocompatibility [32]. Its LCST, in aqueous solution, is located at around 50 °C, at neutral pH, but it can be easily modulated by adjusting the pH of the aqueous solution, the hydrophilic/hydrophobic balance depending on the protonation degree of the tertiary amine groups. The LCST of PDMAEMA, at a pH around 7.0, is much higher than the body temperature, which is not recommended for thermoresponsive DDSs; however, it could be decreased by the copolymerization of DMAEMA with other acrylic monomers, such as acrylamide, when the formation of hydrogen bonds between amide and *N*,*N*-dimethylamino groups increased the copolymer hydrophobicity by screening the hydrophile groups [33]. The presence of tertiary amine groups allows cross-linking with various multifunctional compounds by nucleophilic substitution. Among the available cross-linkers, those derived from natural resources are of great interest for biomedical applications. The volume phase transition temperature (VPTT) of PDMAEMA hydrogels could be decreased by increasing the cross-linker content, which has as consequences the increase in the cross-linking density, the decrease in the mesh size and swelling, and finally an increase in the hydrophobicity of the gel [24].

The role of the inclusion complexes in enhancing the CCM solubility and for the preparation of novel systems for sustained delivery of CCM has been evaluated [34,35,36]. Thus, carboxymethylcellulose-γ-hydroxypropyl cyclodextrin (CD) complexes have been prepared and encapsulated into CS nanoparticles by Popat et al., who have found that CD not only enhanced CCM solubility but also increased its cellular uptake [34]. Incorporation of an inclusion complex of CCM with β-CD into almond gum nanofibers showed a higher solubility and a higher release of CCM in comparison with nanofibers having only CCM [36]. Cryogels, by their particular properties, have been widely used for decades as DDS for various drugs [24,25,37,38,39,40,41,42,43], and also as highly efficient sorbents for the removal of priority pollutants [44,45,46,47]. These materials have been implemented in a wide range of biological, medical, pharmaceutical, and environmental applications due to their outstanding properties such as biocompatibility, hydrophilicity and lack of toxicity. Therefore, the main goals of this work were the synthesis and characterization of novel composite cryogels prepared in two steps: (i) semi-IPN cryogels were prepared first by the entrapment of linear PDMAEMA into PAAm as a primary network; (ii) cross-linking of PDMAEMA free chains with a reactive derivative of β-cyclodextrin (β-CD), i.e., monochlorotriazinyl-β-CD sodium salt (MCT-β-CD), thus generating the secondary network. MCT-β-CD has been intensively used last decade in the textile industry for dyeing and finishing polyester and cellulosic textiles [48,49,50,51,52,53,54], here being used for the first time as a cross-linker in the preparation of novel IPN cryogels that have as destination their use as DDS in the controlled delivery of CCM. The composite cryogels were characterized in terms of the chemical structure (by FTIR and EDX spectra), the internal morphology (SEM) and swelling behavior. The swelling kinetics and equilibrium water uptake as a function of pH were measured by conventional gravimetric method. CCM release profiles were investigated by UV-Vis spectrometry as a function of the matrix composition and pH. To the best of our knowledge, composite cryogels similar to those investigated in this paper have not been previously described in the literature.

## 2. Results and Discussion

**Figure 1:**
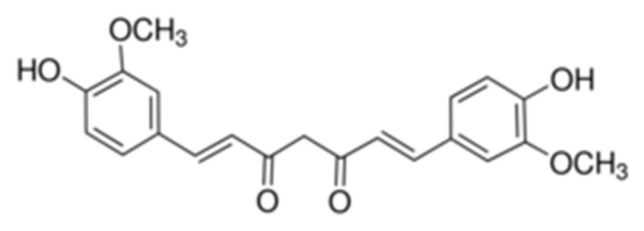
Chemical structure of CCM.

The chemical name of CCM is 1,7-bis(4-hydroxy-3-methoxyphenyl)-hepta-1,6-diene-3,5-dione, its structure being presented in Figure 1.

CCM presents a keto-enol tautomery, the diketo tautomer being stabilized in the presence of water [55].

**Figure 2:**
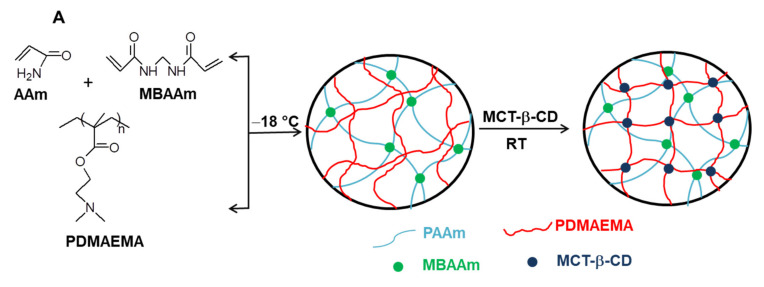

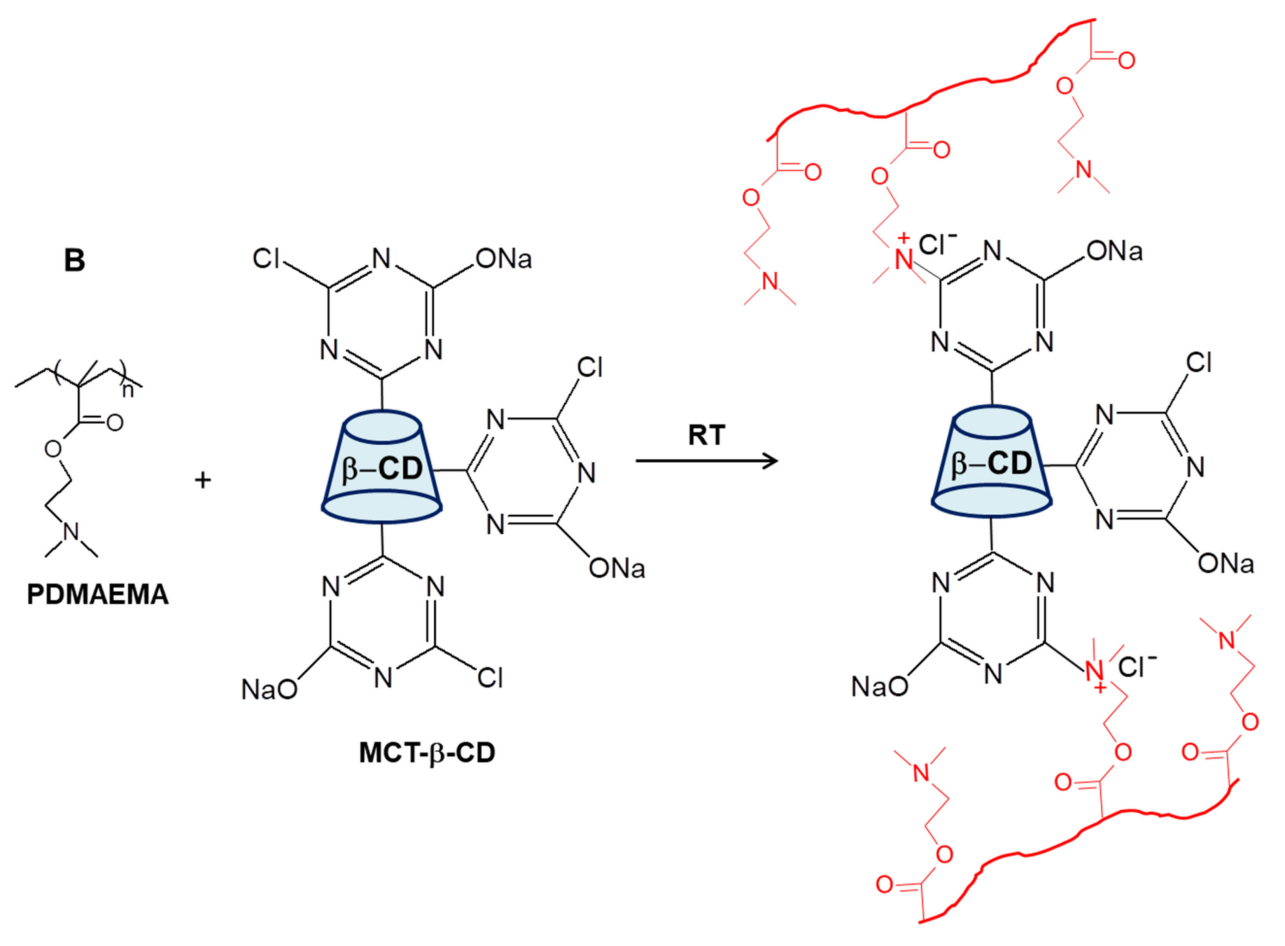
Schematic representation of the synthesis of semi-IPN and IPN cryogels (**A**); cross-linking reaction of PDMAEMA with MCT-β-CD (**B**).

By conducting the cross-linking polymerization of AAm in the presence of linear PDMAEMA in an apparently frozen reaction system, at −18 °C, semi-IPN cryogels were generated. The frozen zones (ice crystals) of the reaction system act as a template during gelation, leading to a porous structure by thawing at the end of the gel preparation [37,38,39,40,41,42,43,44,45,46,47]. The presence of linear PDMAEMA chains containing reactive tertiary amino groups gives the possibility to prepare IPN cryogels by the reaction with multifunctional compounds. Thus, the reaction of PDMAEMA with MCT-β-CD could conduct either to the grafting of MCT-β-CD or to the cross-linking of PDMAEMA generating the second network, depending on the molar ratio between the reactive groups. The reaction is a nucleophilic substitution, which takes place between the reactive chlorine atoms of MCT-β-CD and the tertiary amine groups of PDMAEMA. Schematic presentation of the strategy used for the preparation of PAAm/PDMAEMA IPN cryogels in this work is presented in Figure 2.

### 2.1. Synthesis of Composite Cryogels Based on Polyacrylamide and Poly(N,N-dimethylaminoethyl methacrylate)

As can be seen in Figure 2, the synthesis of multicomponent macroporous cryogels was performed by cross-linking radical cryopolymerization of AAm with *N*,*N’*-methylenebisacrylamide (BAAm), in the presence of PDMAEMA, when a semi-IPN hydrogel having PAAm as a matrix and PDMAEMA as entrapped polymer resulted [37,56]. PDMAEMA with three molar masses (PDMAEMA50, PDMAEMA85 and PDMAEMA250, Table 1), prepared according to the previously published conditions [57], were used to evaluate the influence of the PDMAEMA molar mass and of the polymer concentration on the gel fraction yield (GFY) in the synthesis of semi-IPN gels, and on the properties of composite cryogels.

The synthesis conditions of semi-IPN cryogels are presented in Table 2. In all syntheses, the concentration of the primary network (cross-linked PAAm) was kept constant at 5 wt.%, while the concentration of PDMAEMA varied in the range 0.225–2.5 wt.%.

As can be seen in Table 2, the GFY was higher in the case of PDMAEMA250 than in the case of PDMAEMA85, and also increased with the increase in the cross-linking degree from 1:80 up to 1:40. The values of GFY were lower in the case of the samples prepared by unidirectional freezing (s-IPN250.5 and s-IPN85.3).

### 2.2. Morphological and Chemical Characterization of the Semi-IPN Cryogels

**Figure 3:**
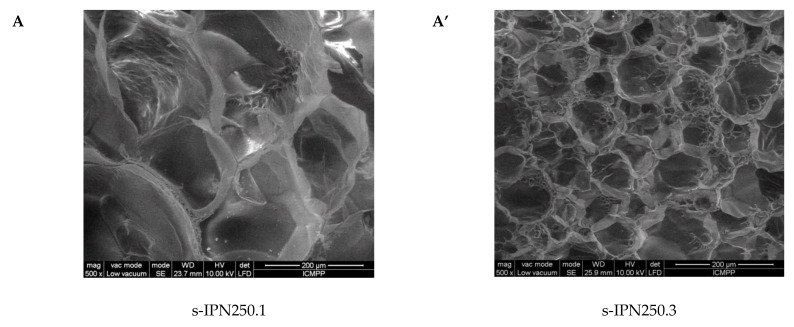

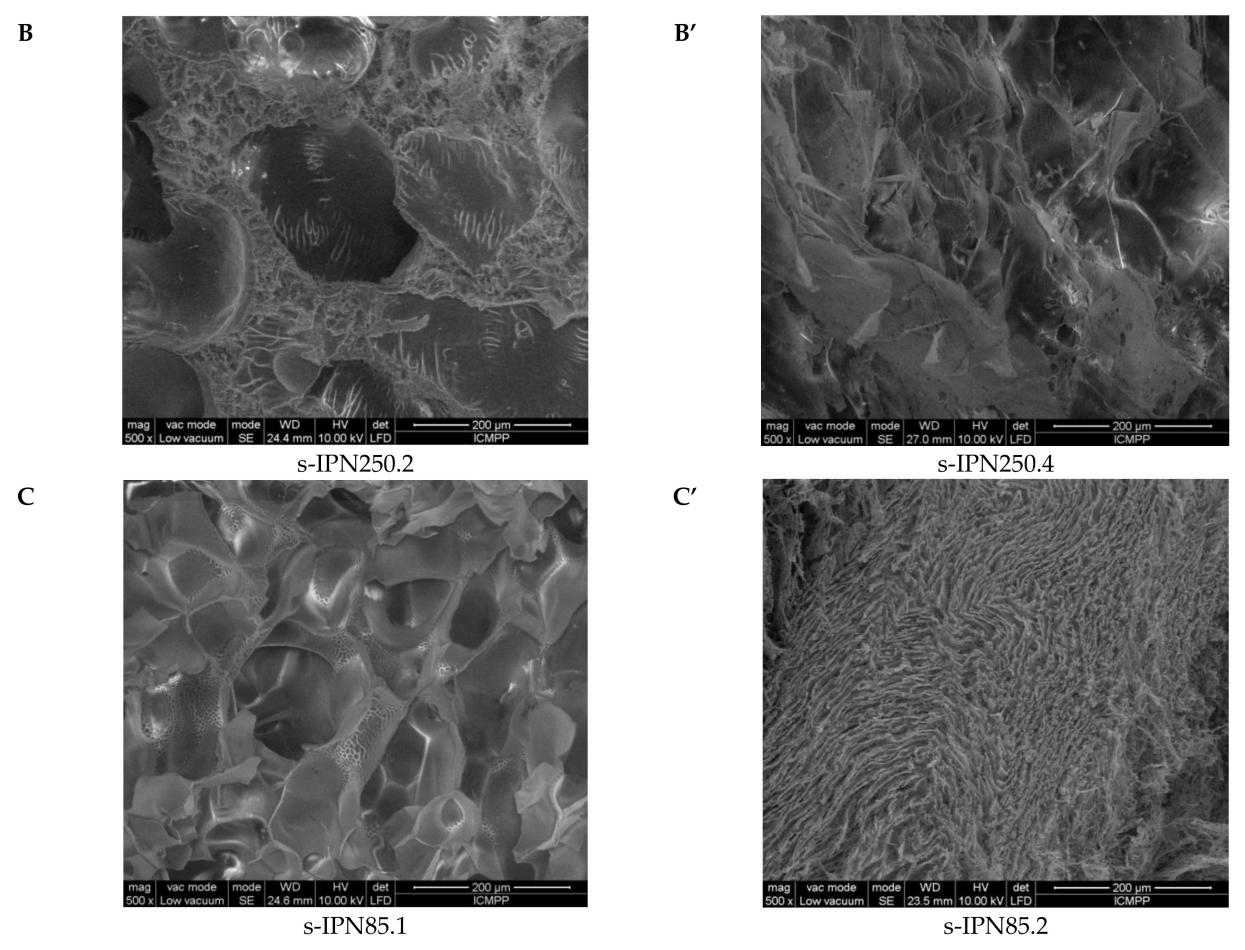
Cross-sectional SEM micrographs of the semi-IPNs with PDMAEMA250 (images **A**, **A’**, **B**, and **B’**) and PDMAEMA85 (images **C** and **C’**), at a cross-linking ratio of PAAm of 1:80 (images **A**, **B**, **C**) and of 1:40 (images **A’**, **B’**, **C’**). (The composition of the samples can be found in Table 2 following the sample code).

Interior morphology of the semi-IPN composite cryogels as a function of the cross-linking ratio of PAAm and the molar mass of PDMAEMA is presented in Figure 3.

The images in Figure 3 clearly show that at a cross-linking ratio of 1:40 (s-IPN250.3, s-IPN250.4, and s-IPN85.2), the interior of the cryogels is much more compact than that of the cryogels prepared at a cross-linking ratio of 1:80 (s-IPN250.1, s-IPN250.2, and s-IPN85.1). The influence of PDMAEMA molar mass is also visible, the lower molar mass (PDMAEMA85) leading to a higher compactness than the polycation with the higher molar mass (PDMAEMA250), at the same concentration (image of s-IPN85.2 compared with that of s-IPN250.4). These differences are attributed to the possibility of the polycation chains having a lower contour length to adopt more coiled conformations than those with a higher contour length. SEM images of two semi-IPN cryogels synthesized by unidirectional freezing (UF) [58] are presented in Figure S1, Supplementary Materials. The influence of the UF technique on the morphology of cryogels is apparent in both the cases of composite cryogel synthesized with PDMAEMA250 (s-IPN250.5) and with PDMAEMA85 (s-IPN85.3). As can be seen, a heterogeneous morphology consisting of randomly distributed polyhedral pores is visible in images s-IPN250.3 and s-IPN85.2 (Figure 3), while an oriented structure, with microchanneled structures arranged along the freezing direction, are obvious for the corresponding samples prepared by UF strategy (the images of the samples s-IPN250.5 and s-IPN85.3, Figure S1).

The pore sizes evaluated from the SEM micrographs by the ImageJ 1.48v software, on three images, the number of pores measured per image being 15 [24,25,59], are presented in Table 3.

As Table 3 shows, the increase in the cross-linking ratio conducted to the decrease in the average pore diameter, as expected, in both the cases of composites prepared with PDMAEMA250 (sample s-IPN250.3 compared with sample s-IPN250.1) and PDMAEMA85 (sample s-IPN85.2 compared with sample s-IPN85.1). Polycation molar mass had also an influence on the average pore diameter, this being higher in the case of PDMAEMA250 (sample s-IPN250.2) than in the case of PDMAEMA85 (sample s-IPN85.1), at the same content in polycation and the same cross-linking degree. The explanation would be that the polymer chains of the polycation with the lower molar mass (PDMAEMA85) could be more compact arranged compared with those of the polycation having a much higher molar mass (PDMAEMA250). The synthesis strategy also had an influence on the pore size, this being higher when the UF was adopted as the synthesis method (samples s-IPN250.5 compared with s-IPN.3, and s-IPN85.3 compared with s-IPN85.2).

Some FTIR spectra of semi-IPN cryogels are presented in Figure S2. The strong band located in the range 3440–3450 cm^−1^ is attributed to the axial stretching of O-H and N-H and to the hydrogen bonds. The bands located at 2930 cm^−1^, 2862 cm^−1^, and at around 2772 cm^−1^ indicate typical stretch vibrations of C-H in CH_2_ and -CH_3_ groups in PDMAEMA and PAAm. The bands at around 1663 cm^−1^, 1610 cm^−1^ and 1317 cm^−1^ were assigned to the stretching vibrations of C=O in amide groups (amide I), N-H vibration (amide II), and C-N (amide III) [30,60]. The band at 1450 cm^−1^ is attributed to the bending vibration of CH_2_ groups. The band located at 1117 cm^−1^, characteristic to the C-N stretching (secondary amide from BAAm), can be observed in Figure S2. The band at around 770 cm^−1^, visible in all spectra, was assigned to bending vibration of N-H groups. However, because the content in PDMAEMA was much lower than that of PAAm, the intense bands of PAAm screened the band characteristic to the ester groups in PDMAEMA, situated at 1728 cm^−1^.

### 2.3. Swelling Kinetics of Semi-IPN PAAm/PDMAEMA Cryogels

**Figure 4:**
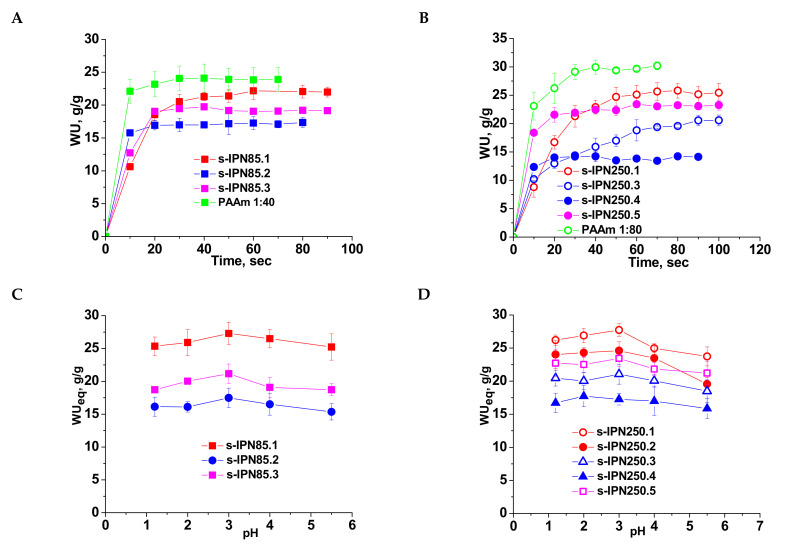
Swelling kinetics of semi-IPN cryogels having PDMAEMA85 (**A**) and PDMAEMA250 (**B**) as linear polymer entrapped in the PAAm matrix, at pH 5.5; WU_eq_ as a function of pH for semi-IPN constructed with PDMAEMA85 (**C**) and PDMAEMA250 (**D**).

Swelling behavior of the semi-IPN cryogels gave information on the influence of the cross-linking ratio in the PAAm network, and on the molar mass and content of PDMAEMA entrapped therein. The values of the water uptake (WU), evaluated with Equation (2) (Section 3.2.4.) as a function of time (swelling kinetics) are presented in Figure 4A (for PDMAEMA85) and 4B (for the composites prepared with PDMAEMA250). The swelling kinetics were compared with those of the single PAAm network having the same cross-linking ratio. The swelling behavior of all semi-IPN cryogels support the specific swelling kinetics of this type of porous materials, i.e., very fast swelling. However, a clear influence of the cryogel composition can be seen. 

Thus, the highest values of WU at equilibrium were found at a cross-linking ratio of 1:80, and these are 22 g/g (composite s-IPN85.1) and 25.4 g/g (composite s-IPN250.1), when the polycation entrapped was PDMAEMA85 or PDMAEMA250, respectively. These values are lower than those found for PAAm cryogel having the same cross-linking ratio (Figure 4A,B, PAAm 1:80, and PAAm 1:40) because of the presence of PDMAEMA chains. The values of WU at equilibrium at a cross-linking ratio of 1:40 and the same content in polycation were 17.35 g/g for s-IPN85.2 and 14.2 g/g for s-IPN250.4. The synthesis technique had also an influence on the WU at equilibrium (WU_eq_), demonstrated by the samples s-IPN85.3 and s-IPN250.5, which have been prepared by UF technique, higher values of WU_eq_ being observed for these samples compared with the corresponding samples prepared by simple cryogelation. Thus, for the sample s-IPN85.3 the value of WU_eq_ was 19.2 g/g, compared with 17.35 g/g found for s-IPN85.2, and for the sample s-IPN250.5 the value of WU_eq_ was 23.2 g/g, compared with 20.6 found for s-IPN250.3. The higher values of WU_eq_ found in the case of the composites prepared by UF can be attributed to the microchanneled morphology, which allows the adsorption of a higher amount of water at equilibrium.

The influence of pH on the equilibrium WU (WU_eq_) is presented in Figure 4C (for the semi-IPN prepared with PDMAEMA85) and Figure 4D (for the semi-IPN prepared with PDMAEMA250). The presence of polycation inside the semi-IPN cryogels is supported by the profile of the curves, the values of WU increasing with the decrease in pH from 5.5 down to pH 3, followed by a slow decrease for all samples. This behavior is attributed to the protonation of tertiary amine groups of PDMAEMA, making the composite more hydrophilic than the initial one. The excess of H^+^ has as a consequence the screening of the positive charges on the polycation chains thus conducting to a lower decrease in the swelling capacity below pH 3. The order of the WU_eq_ values is similar with that found when the swelling kinetics were investigated (Figure 4A,B).

As can be seen in Table S1, element analysis by EDX spectra gives further information on the presence of PDMAEMA in the structure of semi-IPN cryogels by the presence of Cl in the s-IPN250.4 and s-IPN85.3 cryogels after their swelling at equilibrium at pH 1.2, the samples being different only by the molar mass of PDMAEMA entrapped in the PAAm network.

### 2.4. Synthesis and Characterization of IPN cryogels

From the characterization of semi-IPN cryogels having PDMAEMA85 and PDMAEMA250 as entrapped polycation, it was observed that a lower molar mass of polycation and a cross-linking ratio of 1:40 led to a more compact morphology of semi-IPN cryogel. These results suggested that a lower molar mass of PDMAEMA would be suitable to design semi-IPN having a higher concentration in polycation, and a higher number of tertiary amine groups, which could constitute the precursor for the synthesis of IPN cryogels in a sequential manner using MCT-β-CD as a cross-linker (Figure 2). Therefore, for the synthesis of semi-IPN cryogels with a higher content in PDMAEMA, PDMAEMA50 (Table 1) was used, the synthesis conditions being presented in Table 2.

**Figure 5:**
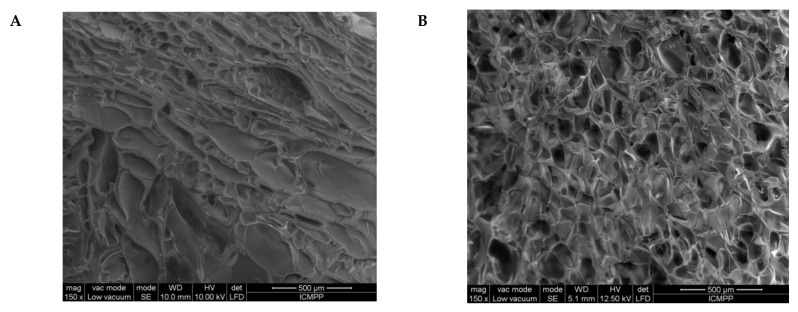

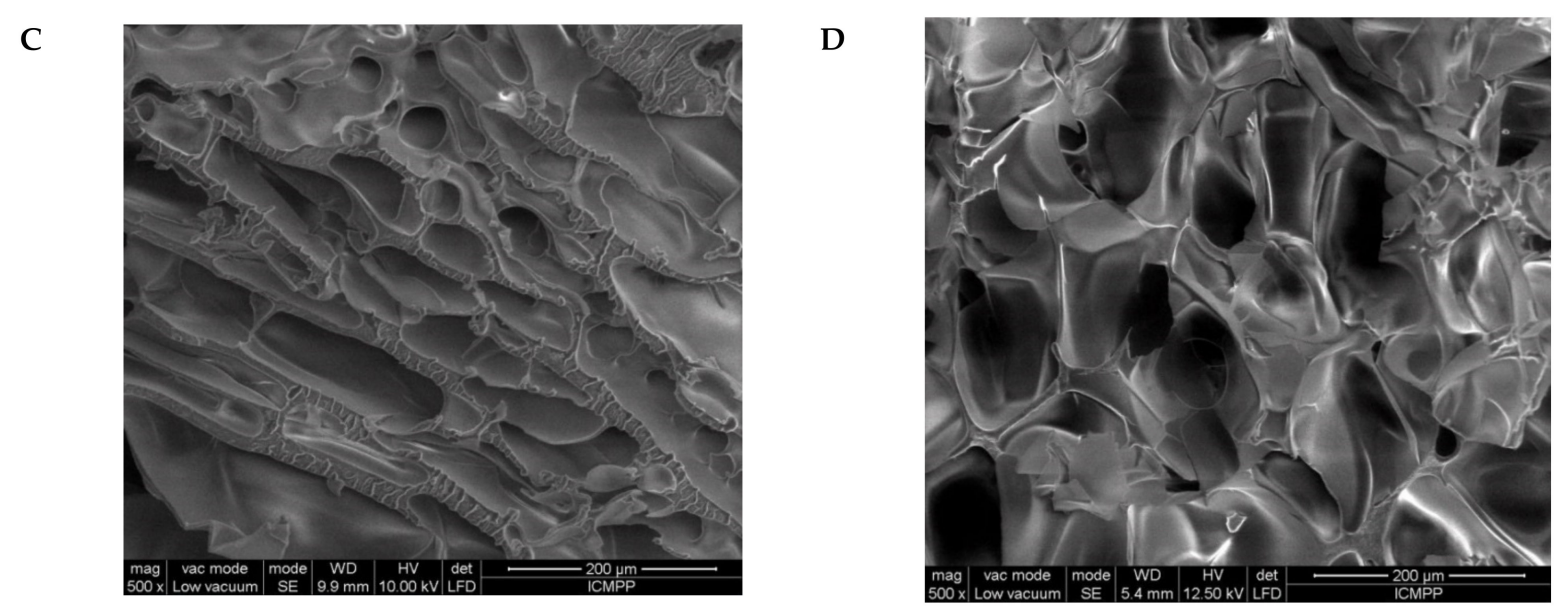
SEM images of the s-IPN50 before (**A**,**C**) and after (**B**,**D**) the reaction with MCT-β-CD, at a magnification of 150× (**A**,**B**) and 500× (**C**,**D**).

The first information about the changes which took place during the preparation of IPN cryogels were given by the examination of SEM images before and after the post-cross-linking of PDMAEMA with MCT-β-CD (Figure 5). As Figure 5 shows, the cryogel morphology dramatically changed after the reaction of PDMAEMA with MCT-β-CD, the interconected pores being visible after this step due to the fact that part of PDMAEMA, the chains non-cross-linked with MCT-β-CD, was removed during the washing steps after cross-linking and also due to the formation of IPN cryogel.

**Figure 6:**
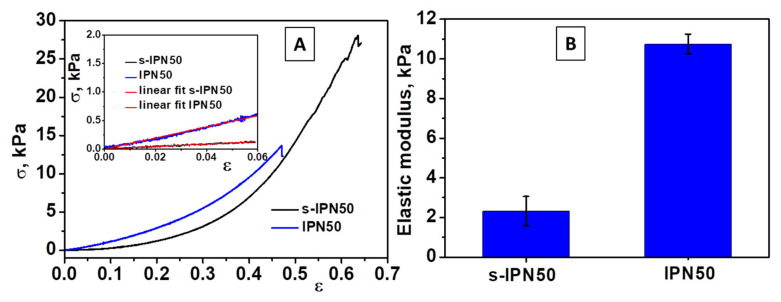
(**A**) Stress−strain curves obtained upon uniaxial compression onto s-IPN50 and IPN50 cryogels (inset: the linear dependence of stress−strain curves from which the elastic moduli were determined); (**B**) The values of modulus of elasticity of s-IPN50 and IPN50 cryogels.

Compression tests were applied on swollen cryogels using a cross-head speed of 1 mm min^−1^ and a force of 50 N up to fracture. The compressive Young’s modulus of s-IPN50 and IPN50 cryogels was determined from the slope of the linear part of the stress−strain curves in accordance with the procedure already reported for triple-network cryogels consisting of chitosan/poly(ethyleneimine)/PDMAEMA [47] and silk fibroin cryogels [61]. Typical stress−strain curves for s-IPN85.2 and IPN50 samples can be seen in Figure 6.

The s-IPN50 cryogel was mechanically stable and sustained a compression of 62% (Figure 6A), while the IPN50 cryogel exhibited high toughness and fractured at 44% compression (Figure 6A, inset). However, the compressive modulus of IPN50 cryogel increased (Figure 6B) with the formation of the second network. The IPN50 cryogel exhibited an elastic modulus of five-times higher than that of the s-IPN50 network. Thus, a dense and stiff network was generated by cross-linking of poly(*N*,*N*-dimethylaminoethyl methacrylate) chains with MCT-β-CD.

**Figure 7:**
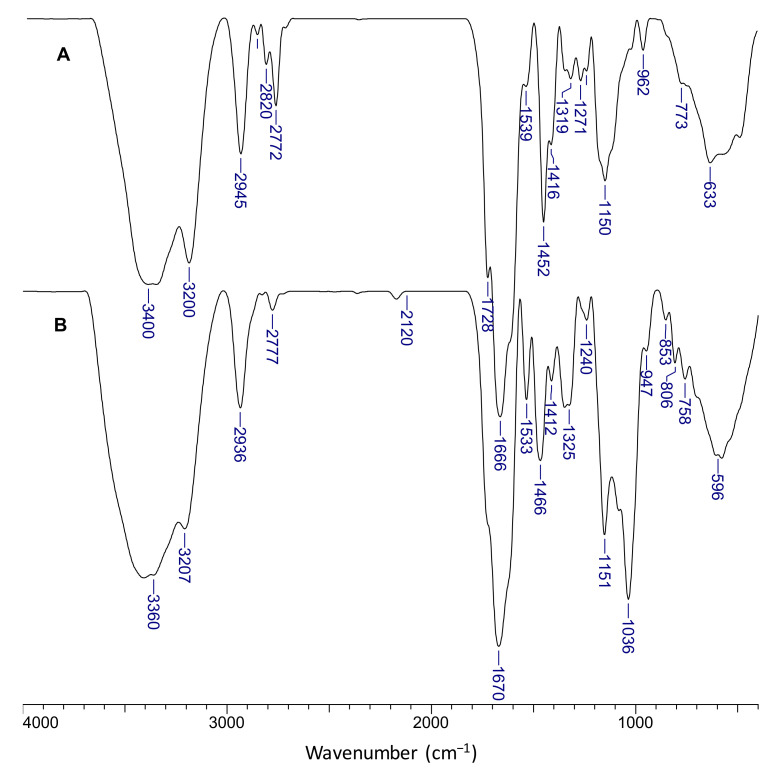
FTIR spectra of the s-IPN50 cryogel (**A**) and the corresponding IPN cryogel (**B**).

The chemical structure of the composite cryogels was investigated by FTIR spectroscopy. Figure 7 presents the spectra of s-IPN50 (A) and IPN obtained after cross-linking with MCT-β-CD (B).

The presence of PDMAEMA is supported by the bands located at 1728 cm^−1^ corresponding to C=O in ester group. The bands at 2945 cm^−1^, 2820 cm^−1^, and at around 2772 cm^−1,^ visible in spectrum A, indicate stretch vibrations of C-H in CH_2_ and -CH_3_ groups in PDMAEMA and PAAm; the presence of these groups in the IPN cryogel (spectrum B) is supported by the bands at 2936 cm^−1^ and 2777 cm^−1^. The bands located at 1666 cm^−1^ and 1319 cm^−1^ in spectrum A were attributed to the bending vibration of C=O (amide I) and C-N bonds (amide III) in PAAm, and the band at 1452 cm^−1^ was attributed to the bending vibrations of CH_2_ groups. The characteristic bands for amide groups in the FTIR spectrum of IPN cryogel (spectrum B) can be seen at 1670 cm^−1^ (amide I) and 1325 cm^−1^ (amide III). The shoulder located at around 1610 cm^−1^ in both spectra is attributed to N-H bonds in amide (amide II). The band located at 1036 cm^−1^ and the peak situated at 853 cm^−1^ are attributed to the C-O-C in glucose units, and to C-O-C of the CD ring [62]. The presence of triazinyl cycle is supported by the peak located at 806 cm^−1^ [63].

### 2.5. Loading and Release of CCM from PAAm/PDMAEMA Composite Cryogels

Some composite cryogels were selected for loading with CCM by the sorption/solvent evaporation technique, the loading being presented in Table 4. Furthermore, the IPN cryogel prepared by cross-linking of PDMAEMA50 with MCT-β-CD, just after the synthesis of s-IPN50 (IPN50), was evaluated as a potential carrier of CCM.

**Figure 8:**
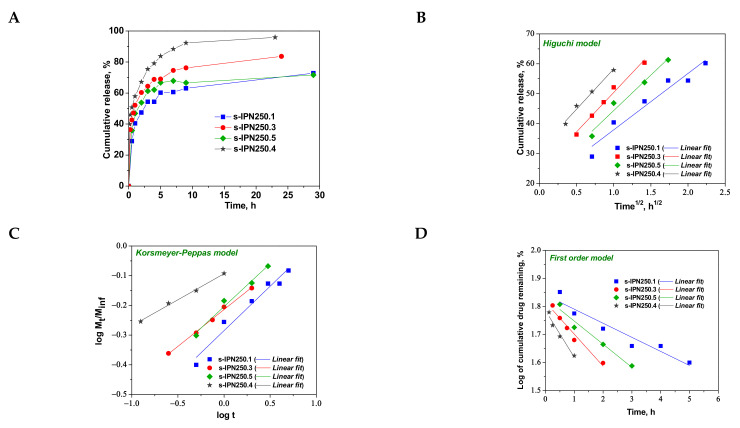
Cumulative release of CCM at pH 3.0, and 37 °C, from semi-IPN with PDMAEMA250 entrapped in PAAm (**A**); fitting of kinetic models: Higuchi (**B**), Korsmeyer–Peppas (**C**), and first order (**D**) models.

**Figure 9:**
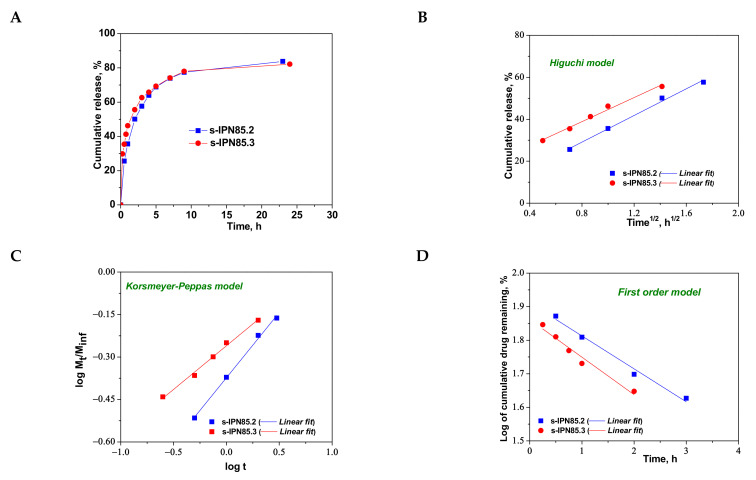
Cumulative release of CCM at pH 3.0, and 37 °C, from semi-IPN with PDMAEMA85 entrapped in PAAm (**A**); fitting of kinetic models: Higuchi (**B**), Korsmeyer–Peppas (**C**), and first order (**D**) models.

It has been reported that the release of CCM from many systems was more effective in an acidic environment (gastric pH) [6,9,15,17,18]. In addition to this, the semi-IPN cryogels having PDMAEMA chains entrapped in PAAm matrix exhibited the maximum WU_eq_ at pH 3, this being attributed to the protonation of polycation having as a consequence the increase in the hydrophilicity and possible conformational changes (Figure 4C,D). Therefore, for in vitro release of CCM at 37 °C, pH 3 was chosen in this study, the cumulative release values as a function of release duration being plotted in Figure 8A and Figure 9A.

The release of CCM from the semi-IPN cryogels was carried out at pH 3.0, containing 5 wt.% Tween 80 to solubilize the released CCM, as mentioned in other papers [7,8].

As can be seen in Figure 8, the maximum cumulative release was found for s-IPN250.4, after 10 h. It seems that the CCM release was faster when the cross-linking ratio of the primary network was higher, i.e., when the carrier is less hydrophilic (see Figure 4B). Comparing samples s-IPN250.3 with s-IPN250.4, which differ by the content in PDMAEMA, a faster cumulative release can be observed in the case of composite s-IPN250.4 than in the case of s-IPN250.3. This also suggests that the decrease in the WU of the carrier (Figure 4B) had a positive influence on the CCM release. A small decrease in the cumulative release of CCM can be seen in the case of s-IPN250.5 compared with s-IPN250.3, the former being prepared by UF strategy and having a higher WU than the composite s-IPN250.3. When PDMAEMA with a lower molar mass was used as entrapped polycation (Figure 9), the cumulative release after 10 h was lower than that found in the case of the corresponding composite that differs only in the molar mass of PDMAEMA (s-IPN250.4). This behavior further supports the positive influence of the increase in hydrophobicity on the cumulative release of CCM.

**Figure 10:**
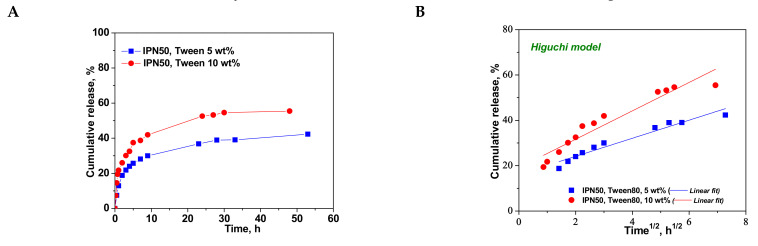

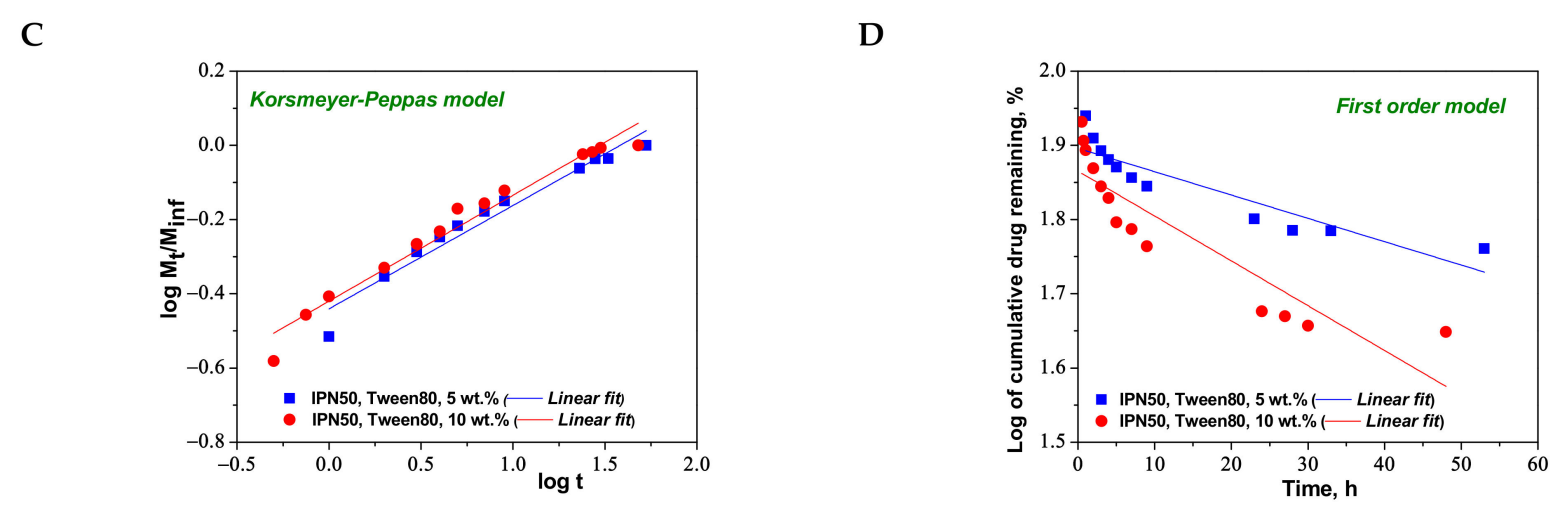
Cumulative release of CCM at pH 3.0, and 37 °C, from IPN50 (**A**); fitting of kinetic models: Higuchi (**B**), Korsmeyer–Peppas (**C**), and first order (**D**) models.

Figure 10 shows that burst-free and sustained release of CCM was found in the case of IPN cryogel. Decrease in the release rate from the IPN as drug carrier is attributed to the two barriers that must be overcome by the CCM molecules, the getting out of the hydrophobic cavity of CD and the more hydrophilic environment generated by the cross-linking of PDMAEMA with MCT-β-CD. Omer et al. have reported a sustained CCM release from the quaternized aminated chitosan nanoparticles, the release rate decreasing with the increase in the quaternization degree, i.e., with the increase in hydrophilic character of the drug carrier [15]. The release rate of CCM has been also decreased by coating nanolyposomes with CS [14]. It has also been reported in the literature that by coating mesoporous silica-based carriers with tannic acid-Fe(III) complex, the sustainability of CCM release increased with the number of coatings [2].

To understand the release mechanism, the experimental data for CCM release were fitted to some kinetic models, Higuchi [64,65], Korsmeyer–Peppas [65,66], and first-order models [9] (Figure 8, Figure 9 and Figure 10), the equations of which are presented in Table S2. The kinetic parameters and the values of *R^2^* are presented in Table 5 for semi-IPN cryogels. The applicability of Higuchi model is based on some assumptions: (i) the initial drug concentration in the carrier is much higher than the drug solubility; (ii) the drug diffusion is unidirectional; (iii) the matrix swelling or dissolution are negligible; (iv) drug particles are much smaller than the system thickness; (v) the drug diffusion coefficient is constant; (vi) sink conditions are constant. As can be seen in Table 5, the values of Higuchi constant (*k_H_*) for semi-IPN cryogels having PDMAEMA250 as polycation entrapped in PAAm network are in the order: s-IPN250.4 > s-IPN250.3 > s-IPN250.5 > s-IPN250.1, and the values of *R^2^* are in the range 0.93 < *R^2^* < 0.98, which supports diffusion as the mechanism of CCM release. The values of *k_H_* and *R^2^* obtained for composites s-IPN85.2 and s-IPN85.3 (Table 5) also support the drug diffusion as the mechanism of CCM release.

Korsmeyer–Peppas model [65,66] is usually used to predict the drug release mechanism from polymeric systems [18,66]. Exponent *n_r_* gives indication on the drug release mechanism and can be evaluated taking into account only the values of fractional drug release, M_t_/M_∞_ < 0.6. Thus, for cylinder geometry *n_r_* = 0.45 indicates a Fickian diffusion of drug, also known as Case I, and occurs when the rate of diffusion is significantly slower than the polymer chains relaxation; 0.45 < *n_r_* < 0.89 indicates an anomalous or non-Fickian release of drug, and occurs when the rate of diffusion and the polymer chain relaxation are comparable; Case II of transport is suggested when *n_r_* = 0.89. The values of *n_r_* and *k_KP_*, evaluated from the slopes and intercepts of the plots of log (M_t_/M_∞_) versus log t for the release of CCM from semi-IPN cryogels (Figure 8C and Figure 9C), are presented in Table 5. As can be observed, the values of *n_r_* are lower than 0.45 for all systems and this suggests a pseudo-Fickian diffusion of drug. Furthermore, the high values of *R^2^* support the applicability of this kinetic model to describe the CCM release from these systems. First-order kinetic model shows that the drug release depends both on the drug concentration and time (Figure 8D and Figure 9D). The high values of *R^2^* found for this kinetic model also support its applicability in the kinetic analysis of CCM release.

The kinetic parameters for the release of CCM from the IPN50 are presented in Table 6.

The values of *k_H_* found for the CCM release from IPN50 (Table 6) were much lower than those found for semi-IPN as matrix for CCM delivery probably because the presence of the second network constitutes a barrier in the release of drug. The high values of *R^2^* in Korsmeyer–Peppas equation indicate the applicability of this model in the evaluation of the release mechanism of CCM. The value of *n_r_* of around 0.28 indicates a pseudo-Fickian diffusion of CCM. The much lower values of *R^2^* found for the first-order model suggest that this kinetic model is not suitable to describe the CCM release from the IPN50 cryogel.

## 3. Materials and Methods

### 3.1. Materials

AAm, BAAm, ammonium persulfate (APS), triethyl amine (TEA) *N*,*N*,*N’*,*N’*-tetramethylethylenediamine (TEMED), DMAEMA, all purchased from Sigma-Aldrich (Chemie GmbH), Schnelldorf, Germany, were used as received. MCT-β-CD with an average degree of substitution of 0.4 (i.e., 2.8 per β-CD) was supplied by Wacker-Chemie (GmbH), München, Germany.

### 3.2. Methods

#### 3.2.1. Synthesis of PDMAEMA

The first step in the preparation of the novel cryogels was the synthesis of PDMAEMA with different molar masses. The synthesis of PDMAEMA was performed by radical polymerization with 2,2’-azo-bis(isobutironitril) (AIBN) as initiator, in toluene as solvent [57]. Polymer has been recovered by precipitation with white spirit. Further purification was performed by dialysis against distilled water for at least three days. The dilute aqueous solutions were concentrated by gentle heating under a vacuum, and the polymer was then recovered by freeze drying with Martin Christ, ALPHA 1-2LD device (Kansas City, MO, Fort Scott, KS, USA) (24 h, at −57 °C and 0.045 mbar). Polymer molar mass was determined by SEC measurements, performed in THF + 2 wt.% TEA, at 35 °C, using a Polymer Laboratories GPC, equipped with PL-EMD 950 Evaporative Mass Detector (Polymer Laboratories Ltd., Shopshire, UK).

#### 3.2.2. Synthesis of Semi-IPN and IPN PAAm/PDMAEMA Cryogels

In the cross-linking copolymerization of AAm with BAAm, TEMED reacted with APS with the generation of sulfate anion-radicals, which initiated the polymerization [67]. The monomer concentration was kept constant at 5 wt.% in all synthesis, the cross-linking ratio of PAAm being either 1 mole BAAm:80 moles AAm or 1 mole BAAm:40 moles AAm (Table 2). The concentration of PDMAEMA in the reaction mixture was varied in the range 0.225–2.5 wt.% (see Table 2). The synthesis of semi-IPN PAAm/PDMAEMA composite cryogel using PDMAEMA85 at a cross-linking ratio of PAAm of 1:40, and a concentration in polycation of 0.445 wt.%, is given as an example. An amount of 0.0445 g PDMAEMA85 was dissolved in 7 mL of distilled water (~12 h). At this solution, 0.4742 g AAm was added. Then, 1 mL aqueous solution of BAAm with a concentration of 0.643 g/25 mL, and 1 mL aqueous solution of TEMED with a concentration of 0.625 mL/25 mL were added and homogenized. The homogeneous reaction mixture was cooled at 0 °C in an ice-water bath, purged with N_2_ for 30 min and then, 1 mL of APS aqueous solution with a concentration of 0.2 g/25 mL was added and the whole mixture was further stirred about 20 s. The reaction mixture was transferred in two syringes of 5 mL, sealed with parafilm and kept 24 h in a cryostat at −18 °C. After thawing at room temperature (about 40 min), the composite cryogels were cut into pieces of about 10 mm, and immersed in water for 48 h, changing water at each four hours (during the day) to wash out the soluble polymers, unreacted monomers and the initiator. Finally, the washing solutions were collected, concentrated up to 200 mL, and analyzed to determine the content of polycation, which left the composite cryogel. The concentration of PDMAEMA, removed from the semi-IPN cryogels, was determined by the polyelectrolyte titration of the washing solutions with a standard aqueous solution of poly(sodium ethylene sulphonate) (concentration of 10^−3^ M) by using the particle charge detector PCD 03, Mütek GmbH, Herrsching, Germany. The average value of PDMAEMA released from the composites was 30 ± 5 wt.%, in the case of the cross-linking degree of 1:80 of the primary network (PAAm), and 25 ± 6 wt.%, in the case of the cross-linking degree of 1:40. Thereafter, the swollen gel samples were frozen in liquid nitrogen, and freeze dried in a Martin Christ, ALPHA 1-2LD device, for 24 h, at −57 °C and 0.045 mbar. To evaluate the GFY, all semi-IPN samples have been further dried under a vacuum in the presence of P_2_O_5_, until the constant weight has been reached. GFY was calculated by Equation (1):

*GFY* (%) = (*W_d_*/*W_m_*) × 100 (1)

where: *W_d_*—weight of dried sample; *W_m_*—weight of monomer, cross-linker, and PDMAEMA remained in the samples after washing steps.

For the synthesis of IPN cryogels, the entrapped polycation was PDMAEMA50 as aqueous solution, at a concentration of 2.5 wt.% in the gel, the cross-linking ratio of PAAm network being 1:40, the other components being identical with those presented above for the synthesis of s-IPN85.2. After thawing at room temperature, the composite cryogels were cut into pieces of about 10 mm, and washed two times each for 20 s with distilled water, with manual stirring, to remove the PDMAEMA on the surface of monoliths. The post-cross-linking of entrapped PDMAEMA50 using MCT-β-CD as aqueous solution with a concentration of 2 wt.% was conducted at room temperature, for 24 h, at a ratio between PDMAEMA and MCT-β-CD of 1:0.5. After cross-linking, the composite cryogels were intensively washed three days at least with distilled water, changing water at intervals of four hours, to remove the soluble components. Finally, the IPN cryogels were frozen in liquid nitrogen, and freeze dried in a Martin Christ, ALPHA 1-2LD device, for 24 h, at −57 °C and 0.045 mbar.

#### 3.2.3. Equipments for Characterization of Cryogels

The structure of the semi-IPN and IPN cryogels was investigated by FTIR spectroscopy with a Bruker Vertex FTIR spectrometer (Bruker, Ettlingen, Germany), resolution of 2 cm^−1^, by the KBr pellet technique, with 5 mg of composite. The samples were scanned in the range of 4000–400 cm^−1^. The interior morphology of the composite cryogels was explored by SEM using an environmental scanning electron microscope (ESEM) (FEI Company, Hillsboro, OR, USA) type Quanta 200, coupled with EDX (SEM-EDX) for determination of the elemental composition.

The mechanical tests were carried out on swollen semi-IPN or IPN cryogels, as monoliths of about 10–12 mm in diameter and 7–11 mm length, at room temperature, using a Shimadzu testing machine (EZ-LX/EZ-SX Series, Kyoto, Japan). A complete contact between the surface of cryogels and the compression plates of the testing machine was ensured by applying an initial force of 0.1 N before performing each analysis. The compressive strain (ε), stress (σ, kPa), and the elastic moduli (G, kPa) were evaluated according to the previously published protocol [47].

#### 3.2.4. Swelling Behavior of the Composite Cryogels

The evaluation of the swelling kinetics of the composite cryogels was performed by conventional gravimetric procedure, by immersing the composite samples in distilled water. The samples were weighed as a function of time, after wiping the excess surface liquid by filter paper. The WU (g/g) was calculated by Equation (2) [24]:

*WU* = (*W_t_* − *W_d_*)/*W_d_* (2)

where: *W_d_* is the weight (g) of the dried cryogel, and *W_t_* is the weight (g) of the hydrated cryogel at time *t*.

#### 3.2.5. Loading and Release of Curcumin

Composite cryogels were loaded with CCM by the sorption-solvent evaporation technique. Solutions of CCM in ethanol with a concentration of 5 mg/mL were prepared first and added to the certain amounts of cryogels as carriers according to the maximum sorption capacity obtained from the investigation of the WU at equilibrium. The samples were kept two hours in closed bottles, at +4 °C, in the dark, for the equilibration of drug sorption. After that, the bottles were opened and kept in the dark for 24 h, for solvent evaporation, and then transferred into a vacuum oven, in the dark, for 24 h. The loading of carriers with CCM was evaluated by weighing the dried samples. The cryogels investigated in this work as carriers for CCM are presented in Table 4.

The in vitro release of CCM was performed in simulated gastric fluid (SGF), at pH 3, by immersing the sample loaded with CCM in 10 mL release medium containing 5 wt.% of Tween 80, if other concentration was not specified. At predetermined time intervals, 1 mL of supernatant were withdrawn and analyzed for the concentration of CCM at λ_max_ of 431 nm [35] using a UV-Vis Spectrophotometer (SPECORD 200 Analytik Jena, Jena, Germany), based on a previously made calibration curve (Figure S3). The removed solution was replaced with an identical volume of fresh releasing solution to keep the volume constant. The cumulative release of CCM was calculated using Equation (3):

% CCM (released) = [(10*C_n_* + Σ*C_n−1_*)/*m_o_*] × 100 (3)

where *C_n_* and *C_n−1_* are the concentrations of CCM (mg L^−1^) in the releasing medium after *n* and *n−1* withdrawing steps; *n* is the number of withdrawing steps of the release medium; *m_o_* is the amount of drug loaded in the sample.

## 4. Conclusions

The influence of the cross-linking degree in PAAm as a primary network, the molar mass and the content of PDMAEMA entrapped in the semi-IPN cryogels on the morphology and swelling properties of semi-IPN cryogels was explored and compared with PAAm as single network cryogels. It was found that the WU decreased by the incorporation of PDMAEMA into the PAAm network. The lowest values of WU and the highest compactness of the cryogel morphology were found when PDMAEMA85 was the entrapped polycation, at the same cross-linking ratio. IPN gels were obtained by the cross-linking reaction of PDMAEMA50, present in s-IPN50 cryogel, with MCT-β-CD. Semi-IPN cryogels and IPN were evaluated as potential delivery systems for CCM. This study indicates that the IPN composite cryogels having PAAm as the first network and PDMAEMA cross-linked with MCT-β-CD as the second network would be a suitable system for the sustained release of CCM in an acidic environment. The semi-IPN cryogels released CCM much faster than IPN cryogel. This strategy could be extended to encapsulate other drugs and to control their release in the gastrointestinal tract.

## Figures and Tables

**Table 1:** PDMAEMA used in the synthesis of semi-IPN cryogels.

Code of PDMAEMA	M_w_, kg mol^−1^	M_w_/M_n_	L_c_^a^, nm
PDMAEMA50	50	2.2	80
PDMAEMA85	85	2	135
PDMAEMA250	250	2.4	398

^a^ polymer contour length = polymerization degree × 0.25 (monomer unit length, nm).

**Table 2:** Synthesis conditions of semi-IPN cryogels having PAAm as primary network and PDMAEMA as entrapped polycation.

Sample Code	PAAm		PDMAEMA		GFY *, %
	Conc., wt.%	Cross-Linking, Mole BAAm:Moles AAm	M_v_, kDa	Conc., wt.%	
s-IPN250.1	5	1:80	250	0.225	82
s-IPN250.2	5	1:80	250	0.45	88
s-IPN250.3	5	1:40	250	0.225	87
s-IPN250.4	5	1:40	250	0.45	86
s-IPN250.5 **	5	1:40	250	0.225	84
s-IPN85.1	5	1:80	85	0.45	80
s-IPN85.2	5	1:40	85	0.45	84
s-IPN85.3 **	5	1:40	85	0.45	82
s-IPN50	5	1:40	50	2.5	-

* Gel fraction yield; ** unidirectional freezing (UF) at −196 °C (liquid nitrogen).

**Table 3:** Morphological characterization of the semi-IPN cryogels as a function of the PDMAEMA molar mass and synthesis conditions (according to Table 2).

Code Sample	Average Pore Diameter, μm
s-IPN250.1	123.92 ± 34.69
s-IPN250.2	151.30 ± 16.56
s-IPN250.3	68.68 ± 16.42
s-IPN250.4	82.55 ± 12.11
s-IPN250.5	80.41 ± 7.72
s-IPN85.1	61.20 ± 8.48
s-IPN85.2	11.42 ± 1.37
s-IPN85.3	45.69 ± 13.22

**Table 4:** Composite cryogels selected for the investigation of CCM release.

Sample Code	s-IPN250.1	s-IPN250.3	s-IPN250.5	s-IPN250.4	s-IPN85.2	s-IPN85.3	IPN50
Loading, mg CCM/g CG	81	54.17	79	74.35	84.6	73.53	62

**Table 5:** Kinetic parameters for the release of CCM from semi-IPN PAAm/PDMAEMA cryogels.

Model Name	Parameters	Sample Code					
		s-IPN250.1	s-IPN250.3	s-IPN250.5	s-IPN250.4	s-IPN85.2	s-IPN85.3
Higuchi	*k_H_*	18.8525	26.3783	23.69.9	26.9382	31.7601	28.7244
	*R^2^*	0.9328	0.9811	0.9606	0.9790	0.9911	0.9848
Korsmeyer–Peppas	*n_r_*	0.2991	0.2479	0.2893	0.17531	0.4607	0.3083
	*k_KP_ (min^−nr^)*	7.5199	8.0734	8.1536	9.1081	6.8844	7.7077
	*R^2^*	0.9567	0.9946	0.9723	0.9937	0.9956	0.9911
First order	*k_1_*	−0.0050	−0.1140	−0.0825	−0.1663	−0.0982	−0.1119
	*R^2^*	0.8993	0.9513	0.9495	0.9492	0.9796	0.9594

**Table 6:** Kinetic parameters for the release of CCM from IPN50.

Model Name	Parameters	Sample Code	
		IPN50 Tween 5 wt.%	IPN50 Tween 10 wt.%
Higuchi	*k_H_*	3.9860	6.2642
	*R^2^*	0.9449	0.9126
Korsmeyer–Peppas	*n_r_*	0.2785	0.2857
	*k_KP_ (min^−nr^)*	6.4369	6.5699
	*R^2^*	0.9516	0.9644
First-order	*k_1_*	−0.0032	−0.0060
	*R^2^*	0.8145	0.7964

## Data Availability

Not applicable.

## Supplementary Materials

The following are available online, Figure S1: SEM images of two semi-IPN cryogels prepared by unidirectional freezing in liquid nitrogen having PDMAEMA250 (s-IPN250.5) or PDMAEMA85 (s-IPN85.3) entrapped into PAAm network at a cross-linking ratio of 1:40, Figure S2: FTIR spectra of some semi-IPN cryogels having: (A) PDMAEMA250 (s-IPN250.3 and s-IPN250.4), or (B) PDMAEMA85 entrapped in the PAAm network (sample code is that given in Table 2 in the main text); Figure S3: Calibration curve of CCM in ethanol: pH 3 aqueous solution, 50:50 (*v*/*v*). Table S1: EDX analysis after pH 1.2; Table S2: The mathematical formula of kinetic models.

## Sample Availability

Samples of the semi-IPN PAAm/PDMAEMA composite cryogels are available from the authors.

## Abbreviations

AAm	Acrylamide
APS	Ammonium persulfate
BAAm	*N*,*N’*-Methylenebisacrylamide
CCM	Curcumin
CD	Cyclodextrin
DDS	Drug delivery system
EDX	Energy-dispersive X-ray spectroscopy
GFY	Gel fraction yield
LCST	Lower critical solution temperature
MCT-β-CD	Monochlorotriazinyl-β-cyclodextrin
PAAm	Polyacrylamide
PDMAEMA	Poly(dimethylamonoethyl methacrylate)
s-IPN	Semi-interpenetrating polymer networks
TEMED	*N*,*N*,*N’*,*N’*-tetramethylethylenediamine
WU	Water uptake
UF	Unidirectional freezing
VPTT	Volume phase transition temperature

## References

[1] Tizabi Y., Hurley L.L., Qualls Z., Akinfiresoye L. (2014). Relevance of the Anti-Inflammatory Properties of Curcumin in Neurodegenerative Diseases and Depression. Molecules.

[2] Kim S., Philippot S., Fontanay S., Duval R.E., Lamouroux E., Canilho N., Pasc A. (2015). pH- and glutathione-responsive release of curcumin from mesoporous silica nanoparticles coated using tannic acid–Fe(iii) complex. RSC Adv..

[3] Benameur T., Soleti R., Panaro M.A., La Torre M.E., Monda V., Messina G., Porro C. (2021). Curcumin as Prospective Anti-Aging Natural Compound: Focus on Brain. Molecules.

[4] Mbese Z., Khwaza V., Aderibigbe B.A. (2019). Curcumin and Its Derivatives as Potential Therapeutic Agents in Prostate, Colon and Breast Cancers. Molecules.

[5] Li L., Zhang X., Pi C., Yang H., Zheng X., Zhao L., Wei Y. (2020). Review of Curcumin Physicochemical Targeting Delivery System. Int. J. Nanomed..

[6] AbouAitah K.E.A., Farghali A.A., Swiderska-Sroda A., Lojkowski W., Razin A.-F.M., Khedr M.H. (2016). pH-controlled Release System for Curcumin based on Functionalized Dendritic Mesoporous Silica Nanoparticles. J. Nanomed. Nanotechnol..

[7] Wang J., Wang Y., Liu Q., Yang L., Zhu R., Yu C., Wang S. (2017). Rational Design of Multifunctional Dendritic Mesoporous Silica Nanoparticles to Load Curcumin and Enhance Efficacy for Breast Cancer Therapy. ACS Appl. Mater. Interfaces.

[8] Daryasari M.P., Akhgar M.R., Mamashli F., Bigdeli B., Khoobi M. (2016). Chitosan-folate coated mesoporous silica nanoparticles as a smart and pH-sensitive system for curcumin delivery. RSC Adv..

[9] Alavijeh R.K., Akhbari K. (2020). Biocompatible MIL-101(Fe) as a Smart Carrier with High Loading Potential and Sustained Release of Curcumin. Inorg. Chem..

[10] Dai L., Sun C., Li R., Mao L., Liu F., Gao Y. (2017). Structural characterization, formation mechanism and stability of curcumin in zein-lecithin composite nanoparticles fabricated by antisolvent co-precipitation. Food Chem..

[11] Witika B.A., Makoni P.F., Matafwali S.K., Mweetwa L., Shandele G., Walker R.B. (2021). Enhancement of Biological and Pharmacological Properties of an Encapsulated Polyphenol: Curcumin. Molecules.

[12] Gunathilake T.M.S.U., Ching Y.C., Chuah C.H., Illias H.A., Ching K.Y., Singh R., Nai-Shang L. (2018). Influence of a nonionic surfactant on curcumin delivery of nanocellulose reinforced chitosan hydrogel. Int. J. Biol. Macromol..

[13] Jardim K.V., Palomec-Garfias A.F., Andrade B.Y.G., Chaker J.A., Báo S., Márquez-Beltrán C., Moya S.E., Parize A.L., Sousa M.H. (2018). Novel magneto-responsive nanoplatforms based on MnFe2O4 nanoparticles layer-by-layer functionalized with chitosan and sodium alginate for magnetic controlled release of curcumin. Mater. Sci. Eng. C.

[14] Hasan M., ElKhoury K., Kahn C.J.F., Arab-tehrany E., Linder M. (2019). Preparation, Characterization, and Release Kinetics of Chitosan-Coated Nanoliposomes Encapsulating Curcumin in Simulated Environments. Molecules.

[15] Omer A., Ziora Z., Tamer T., Khalifa R., Hassan M., Mohy-Eldin M., Blaskovich M. (2021). Formulation of Quaternized Aminated Chitosan Nanoparticles for Efficient Encapsulation and Slow Release of Curcumin. Molecules.

[16] Liu F., Li R., Mao L., Gao Y. (2018). Ethanol-induced composite hydrogel based on propylene glycol alginate and zein: Formation, characterization and application. Food Chem..

[17] Gao N., Lü S., Gao C., Wang X., Xu X., Bai X., Feng C., Liu M. (2016). Injectable shell-crosslinked F127 micelle/hydrogel composites with pH and redox sensitivity for combined release of anticancer drugs. Chem. Eng. J..

[18] Wu L., Tian J., Ye X., Fang H., Zhang Z., Xu C., Zhang H. (2020). Encapsulation and Release of Curcumin with the Mixture of Porous Rice Starch and Xanthan Gum. Starch Stärke.

[19] Qi X., Yuan Y., Zhang J., Bulte J.W.M., Dong W. (2018). Oral Administration of Salecan-Based Hydrogels for Controlled Insulin Delivery. J. Agric. Food Chem..

[20] Qi X., Wei W., Shen J., Dong W. (2019). Salecan polysaccharide-based hydrogels and their applications: A review. J. Mater. Chem. B.

[21] Hu X., Wang Y., Zhang L., Xu M. (2020). Construction of self-assembled polyelectrolyte complex hydrogel based on oppositely charged polysaccharides for sustained delivery of green tea polyphenols. Food Chem..

[22] Caka M., Türkcan C., Uygun D.A., Uygun M., Akgöl S., Denizli A. (2017). Controlled release of curcumin from poly(HEMA-MAPA) membrane. Artif. Cells Nanomed. Biotechnol..

[23] Chiaoprakobkij N., Suwanmajo T., Sanchavanakit N., Phisalaphong M. (2020). Curcumin-Loaded Bacterial Cellulose/Alginate/Gelatin as A Multifunctional Biopolymer Composite Film. Molecules.

[24] Dragan E.S., Cocarta A.I. (2016). Smart Macroporous IPN Hydrogels Responsive to pH, Temperature, and Ionic Strength: Synthesis, Characterization, and Evaluation of Controlled Release of Drugs. ACS Appl. Mater. Interfaces.

[25] Dragan E.S., Loghin D.F.A., Cocarta A.-I., Doroftei M. (2016). Multi-stimuli-responsive semi-IPN cryogels with native and anionic potato starch entrapped in poly(*N*,*N*-dimethylaminoethyl methacrylate) matrix and their potential in drug delivery. React. Funct. Polym..

[26] Dinu M.V., Perju M.M., Drăgan E.S. (2011). Composite IPN ionic hydrogels based on polyacrylamide and dextran sulfate. React. Funct. Polym..

[27] Risbud M.V., Bhonde R.R. (2000). Polyacrylamide-Chitosan Hydrogels: In Vitro Biocompatibility and Sustained Antibiotic Release Studies. Drug Deliv..

[28] Ekici S., Saraydin D. (2004). Synthesis, Characterization and Evaluation of IPN Hydrogels for Antibiotic Release. Drug Deliv..

[29] Zohuriaan-Mehr M.J., Omidian H., Doroudiani S., Kabiri K. (2010). Advances in non-hygienic applications of superabsorbent hydrogel materials. J. Mater. Sci..

[30] Dragan E.S., Lazar M.M., Dinu M.V., Doroftei F. (2012). Macroporous composite IPN hydrogels based on poly(acrylamide) and chitosan with tuned swelling and sorption of cationic dyes. Chem. Eng. J..

[31] Rawlinson L.-A.B., Ryan S.M., Mantovani G., Syrett J.A., Haddleton D., Brayden D. (2010). Antibacterial Effects of Poly(2-(dimethylamino ethyl)methacrylate) against Selected Gram-Positive and Gram-Negative Bacteria. Biomacromolecules.

[32] Peng C.-L., Tsai H.-M., Yang S.-J., Luo T.-Y., Lin C.-F., Lin W.-J., Shieh M.-J. (2011). Development of thermosensitive poly(*n*-isopropylacrylamide-*co*-((2-dimethylamino) ethyl methacrylate))-based nanoparticles for controlled drug release. Nanotechnology.

[33] Cho S.H., Jhon M.S., Yuk S.H., Lee H.B. (1997). Temperature-induced phase transition of poly(*N*,*N*-dimethylaminoethyl methac-rylate-co-acrylamide). J. Polym. Sci. Part B Polym. Phys..

[34] Popat A., Karmakar S., Jambhrunkar S., Xu C., Yu C. (2014). Curcumin-cyclodextrin encapsulated chitosan nanoconjugates with enhanced solubility and cell cytotoxicity. Colloids Surf. B Biointerfaces.

[35] Guo S. (2019). Encapsulation of curcumin into β-cyclodextrins inclusion: A review. E3S Web Conf..

[36] Rezaei A., Nasirpour A. (2019). Evaluation of Release Kinetics and Mechanisms of Curcumin and Curcumin-β-Cyclodextrin Inclusion Complex Incorporated in Electrospun Almond Gum/PVA Nanofibers in Simulated Saliva and Simulated Gastrointestinal Conditions. BioNanoScience.

[37] Lozinsky V.I. (2020). Cryostructuring of Polymeric Systems. 55. Retrospective View on the More than 40 Years of Studies Performed in the A.N.Nesmeyanov Institute of Organoelement Compounds with Respect of the Cryostructuring Processes in Polymeric Systems. Gels.

[38] Jain E., Damania A., Shakya A.K., Kumar A., Sarin S.K., Kumar A. (2015). Fabrication of macroporous cryogels as potential hepatocyte carriers for bioartificial liver support. Colloids Surf. B.

[39] Bakhshpour M., Idil N., Perçin I., Denizli A. (2019). Biomedical Applications of Polymeric Cryogels. Appl. Sci..

[40] Dinu M.V., Cocarta A.I., Dragan E.S. (2016). Synthesis, characterization and drug release properties of 3D chitosan/clinoptilolite biocomposite cryogels. Carbohydr. Polym..

[41] Dragan E.S., Cocarta A.I., Gierszewska M. (2016). Designing novel macroporous composite hydrogels based on methacrylic acid copolymers and chitosan and in vitro assessment of lysozyme controlled delivery. Colloids Surf. B Biointerfaces.

[42] Loghin D.F.A., Biliuta G., Coseri S., Dragan E.S. (2017). Preparation and characterization of oxidized starch/poly(*N*,*N*-dimethylaminoethyl methacrylate) semi-IPN cryogels and in vitro controlled release evaluation of indomethacin. Int. J. Biol. Macromol..

[43] Dragan E.S., Dinu M.V. (2019). Polysaccharides constructed hydrogels as vehicles for proteins and peptides. A review. Carbohydr. Polym..

[44] Dragan E.S., Dinu M.V. (2020). Advances in porous chitosan-based composite hydrogels: Synthesis and applications. React. Funct. Polym..

[45] Hu X., Yan L., Wang Y., Xu M. (2021). Ice segregation induced self-assembly of salecan and grapheme oxide nanosheets into ion-imprinted aerogel with superior selectivity for cadmium (II) capture. Chem. Eng. J..

[46] Dragan E.S., Humelnicu D., Dinu M.V. (2019). Development of chitosan-poly(ethyleneimine) based double network cryogels and their application as superadsorbents for phosphate. Carbohydr. Polym..

[47] Dragan E.S., Humelnicu D., Dinu M.V. (2021). Designing smart triple-network cationic cryogels with outstanding efficiency and selectivity for deep cleaning of phosphate. Chem. Eng. J..

[48] Abdel-Halim E.S., Abdel-Mohdy F.A., Al-Deyab S.S., El-Newehy M.H. (2010). Chitosan and monochlorotriazinyl-β-cyclodextrin finishes inprove antistatic properties of cotton/polyester blend and polyester fabrics. Carbohydr. Polym..

[49] Popescu V., Muresan E.I., Grigoriu A.-M. (2011). Monochlorotriazinyl-β-cyclodextrin grafting onto polyester fabrics and films. Carbohydr. Polym..

[50] Sundrarajan M., Rukmani A. (2012). Durable Antibacterial Finishing on Organic Cotton by Inclusion of Thymol into Cyclodextrin Derivative. J. Chem..

[51] Khanna S., Chakraborty J.N. (2017). Optimization of monochlorotriazine β-cyclodextrin grafting on cotton and assessment of release behavior of essential oils from functionalized fabric. Fash. Text..

[52] Elzairy E.R.M., Abdallah W.A., Osman S.M., Fouad M.A.M. (2017). Recent approach for multifunctional finishing of cotton/polyester fabric blends via natural products. Int. J. Text. Sci..

[53] Setthayanond J., Sodsangchan C., Suwanruji P., Tooptompong P., Avinc O. (2017). Influence of MCT-β-cyclodextrin treatment on strength, reactive dyeing and third-hand cigarette smoke odor release properties of cotton fabric. Cellulose.

[54] Bezerra F.M., Lis M.J., Firmino H.B., Da Silva J.G.D., Valle R.D.C.S.C., Valle J.A.B., Scacchetti F.A.P., Tessaro A.L. (2020). The Role of β-Cyclodextrin in the Textile Industry—Review. Molecules.

[55] Manolova Y., Deneva V., Antonov L., Drakalska E., Momekova D., Lambov N. (2014). The effect of the water on the curcumin tautomerism: A quantitative approach. Spectrochim. Acta Part A Mol. Biomol. Spectrosc..

[56] Lozinsky V.I. (2018). Cryostructuring of Polymeric Systems. 50. Cryogels and Cryotropic Gel-Formation: Terms and Definitions. Gels.

[57] Dragan E.S., Dinu I.A. (2011). Solution behavior of progressively quaternized poly(dimethylaminoethyl methacrylate) as a function of charge density. Eur. Polym. J..

[58] Dinu M.V., Přádný M., Drăgan E.S., Michálek J. (2013). Ice-templated hydrogels based on chitosan with tailored porous morphology. Carbohydr. Polym..

[59] Venkataraman R., Das G., Singh S., Pathak L., Ghosh R.N., Venkataraman B., Krishnamurthy R. (2007). Study on influence of porosity, pore size, spatial and topological distribution of pores on microhardness of as plasma sprayed ceramic coatings. Mater. Sci. Eng. A.

[60] Yuan Q., Shah J., Hein S., Misra R. (2010). Controlled and extended drug release behavior of chitosan-based nanoparticle carrier. Acta Biomater..

[61] Ak F., Oztoprak Z., Karakutuk I., Okay O. (2013). Macroporous Silk Fibroin Cryogels. Biomacromolecules.

[62] Ja’Far M.H., Kamal N.N.S.N.M., Hui B.Y., Kamaruzzaman M.F., Zain N.N.M., Yahaya N., Raoov M. (2018). Inclusion of Curcumin in β-cyclodextrins as Potential Drug Delivery System: Preparation, Characterization and Its Preliminary Cytotoxicity Approaches. Sains Malays..

[63] Martel B., Devassine M., Crini G., Weltrowski M., Bourdonneau M., Morcellet M. (2001). Preparation and sorption properties of a β-cyclodextrin-linked chitosan derivative. J. Polym. Sci. A Polym. Chem..

[64] Higuchi T. (1961). Rate of Release of Medicaments from Ointment Bases Containing Drugs in Suspension. J. Pharm. Sci..

[65] Siepmann J., Peppas N.A. (2011). Higuchi equation: Derivation, applications, use and misuse. Int. J. Pharm..

[66] Peppas N.A., Narasimhan B. (2014). Mathematical models in drug delivery: How modeling has shaped the way we design new drug delivery systems. J. Control. Release.

[67] Lozinsky V.I., Morozova S.A., Vainerman E.S., Titova E.F., Shtilman M.I., Belatseva E.M., Rogozhin S.V. (1989). Study of cryo-structuration of polymer systems. VIII. Characteristic features of the formation of crosslinked poly(acryl amide) cryogels under different thermal conditions. Acta Polym..

