# Supplementary material for: Preparation and Characterization of Semi-IPN Cryogels Based on Polyacrylamide and Poly(N,N-dimethylaminoethyl methacrylate); Functionalization of Carrier with Monochlorotriazinyl-β-cyclodextrin and Release Kinetics of Curcumin
Source: Molecules. 2021 Nov 18;26(22):6975. doi: 10.3390/molecules26226975 (PMC8622513; doi:10.3390/molecules26226975)
Supplement: Supplementary file 1 [file molecules-26-06975-s001.zip › molecules-1461008-supplementary.pdf]

molecules

Supplementary Material

Preparation and Characterization of Semi-IPN Cryogels Based on Polyacrylamide and Poly(*N*,*N*-dimethylaminoethyl methacrylate); Functionalization of Carrier with Monochlorotriazinyl-β-cyclodextrin and Release Kinetics of Curcumin

Ecaterina Stela Dragan *, Maria Valentina Dinu, Claudiu Augustin Ghiorghita, Maria Marinela Lazar and Florica Doroftei

Department of Functional Polymers, “Petru Poni” Institute of Macromolecular Chemistry, 700487 Iași, Romania; vdinu@icmpp.ro (M.V.D.); claudiu.ghiorghita@icmpp.ro (C.A.G.); mariperju@icmpp.ro (M.M.L.); florica.doroftei@icmpp.ro (F.D.)

* Correspondence: sdragan@icmpp.ro; Tel.: +40-74-108-8979

Figure S1. SEM images of two semi-IPN cryogels prepared by unidirectional freezing in liquid nitrogen having PDMAEMA250 (s-IPN250.5) or PDMAEMA85 (s-IPN85.3) entrapped into PAAm network at a cross-linking ratio of 1:40.

Figure S2. FTIR spectra of some semi-IPN cryogels having: (A) PDMAEMA250 (s-IPN250.3 and s-IPN250.4), or (B) PDMAEMA85 entrapped in the PAAm network (sample code is that given in Table 2 in the main text).

Figure S3. Calibration curve of CCM in ethanol: pH 3 aqueous solution, 50:50 (v/v).

Table S1. Element analysis of two s-IPN composites after the swelling at equilibrium in pH 1.2.

Table S2. The mathematical formula of kinetic models.

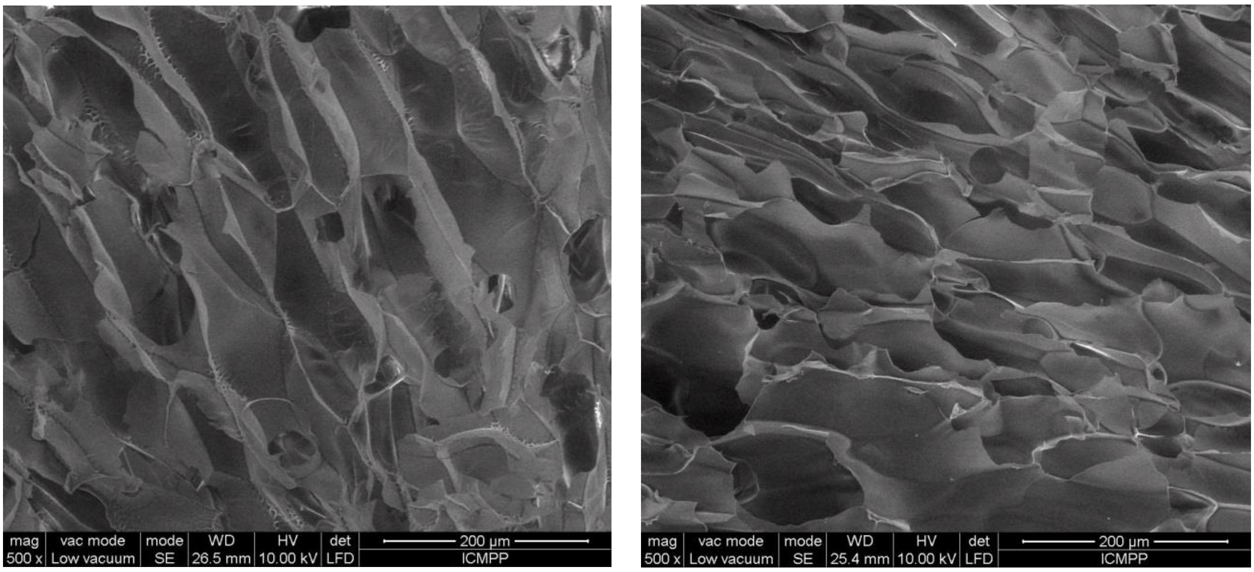

s-IPN250.5 **s-IPN85.3**

Figure S1. SEM images of two semi-IPN cryogels prepared by unidirectional freezing in liquid nitrogen having PDMAEMA250 (s-IPN250.5) or PDMAEMA85 (s-IPN85.3) entrapped into PAAm network at a cross-linking ratio of 1:40.

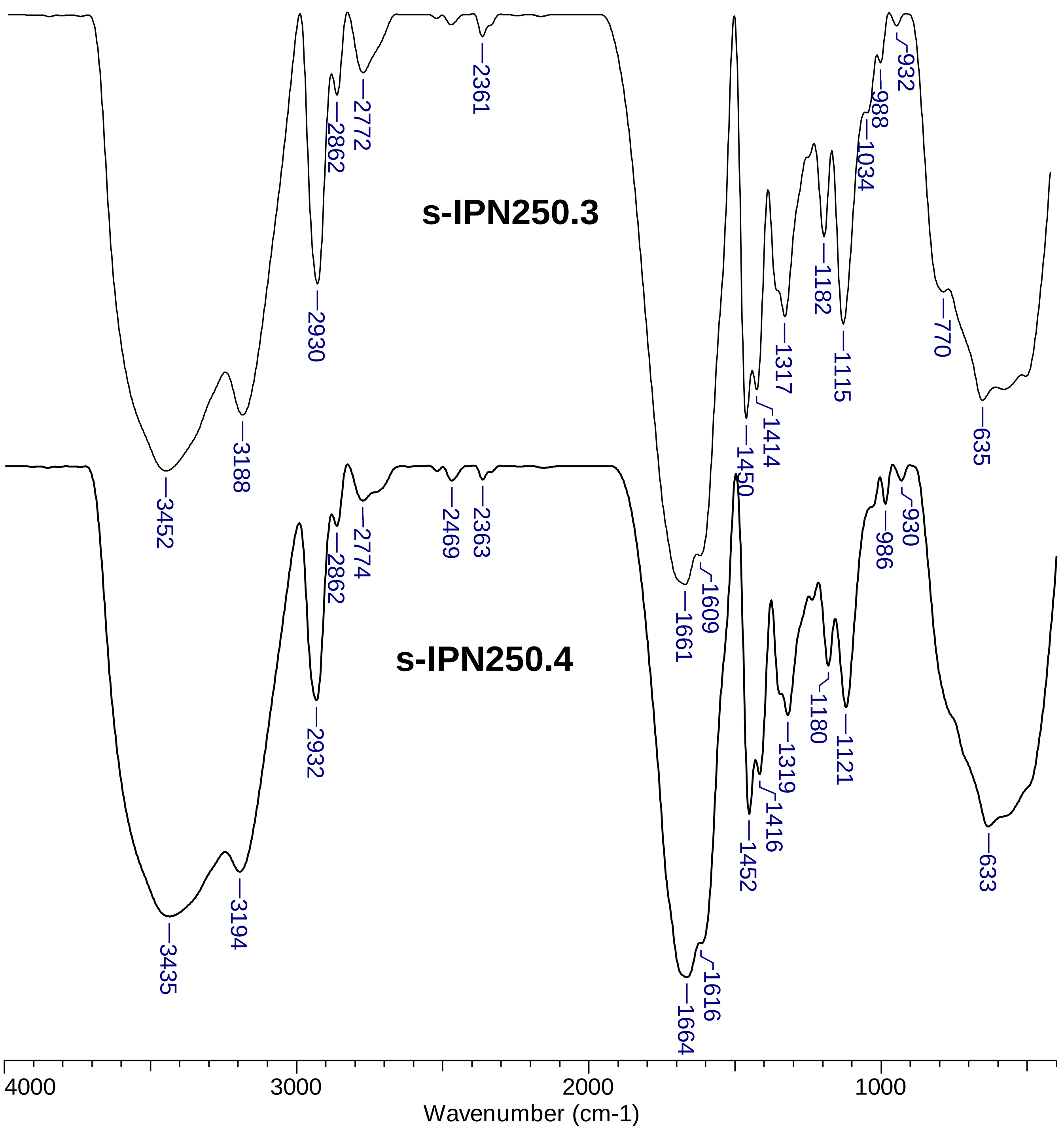

s-IPN250.3
s-IPN250.4
3452
3188
2930
2862
2772
2361
1661
1609
1450
1414
1317
1182
1115
1034
988
932
770
635
3435
3194
2932
2862
2774
2469
2363
1664
1616
1452
1416
1319
1180
1121
986
930
633
4000
3000
2000
1000
Wavenumber (cm-1)

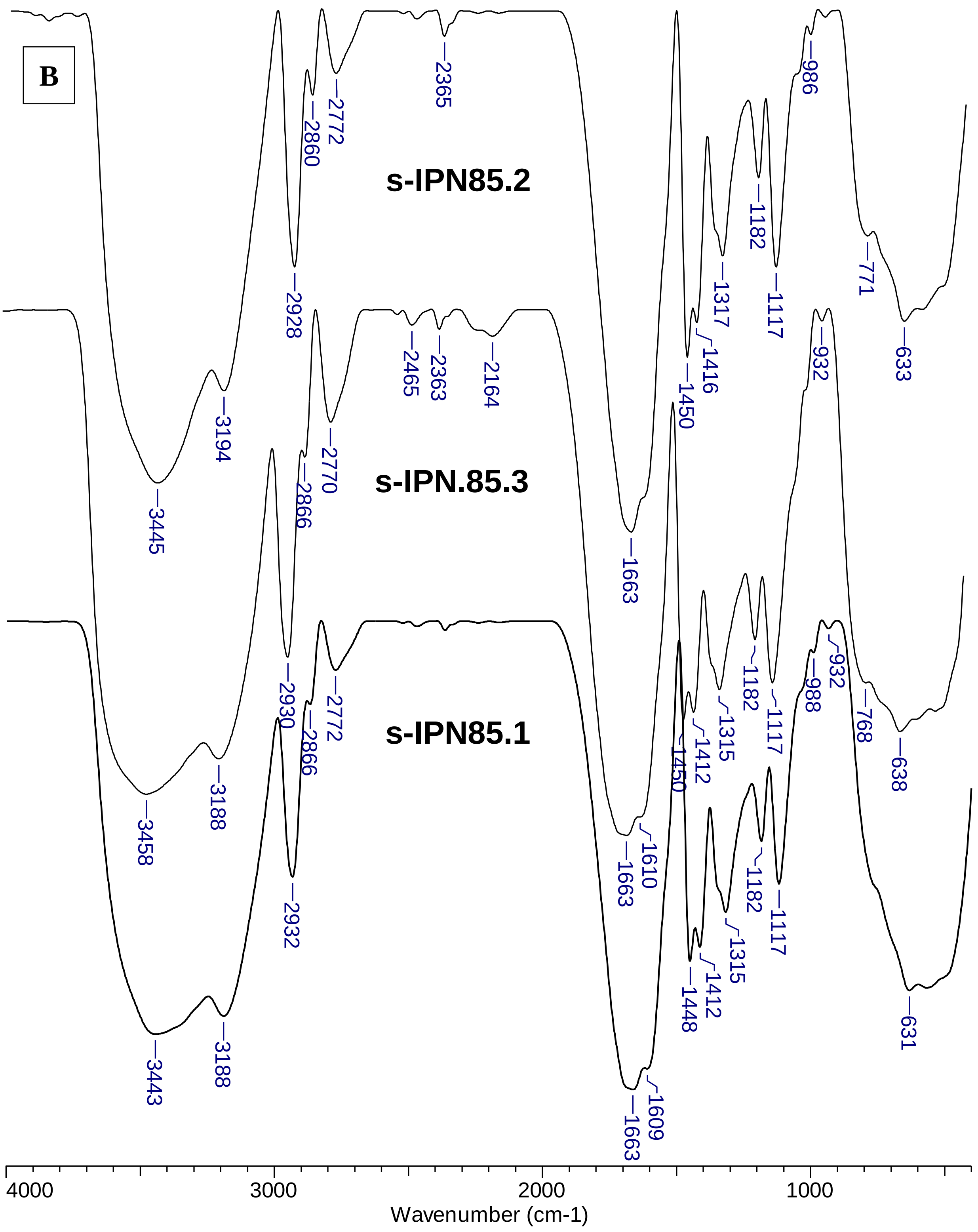

Figure S2. FTIR spectra of some semi-IPN cryogels having: (A) PDMAEMA250 (s-IPN250.3 and s-IPN250.4), or (B) PDMAEMA85 entrapped in the PAAm network (sample code is that given in Table 2 in the main text).

Table S1. Element analysis of two s-IPN composites after the swelling at equilibrium in pH 1.2.

Composite	C %	N %	O %	Cl%
s-IPN250.4	49.93 ± 0.79	21.64 ± 1.02	24.03 ± 1.16	2.97 ± 0.85
s-IPN85.3	51.26 ± 2.64	21.92 ± 1.18	23.48 ± 2.4	1.81 ± 0.68

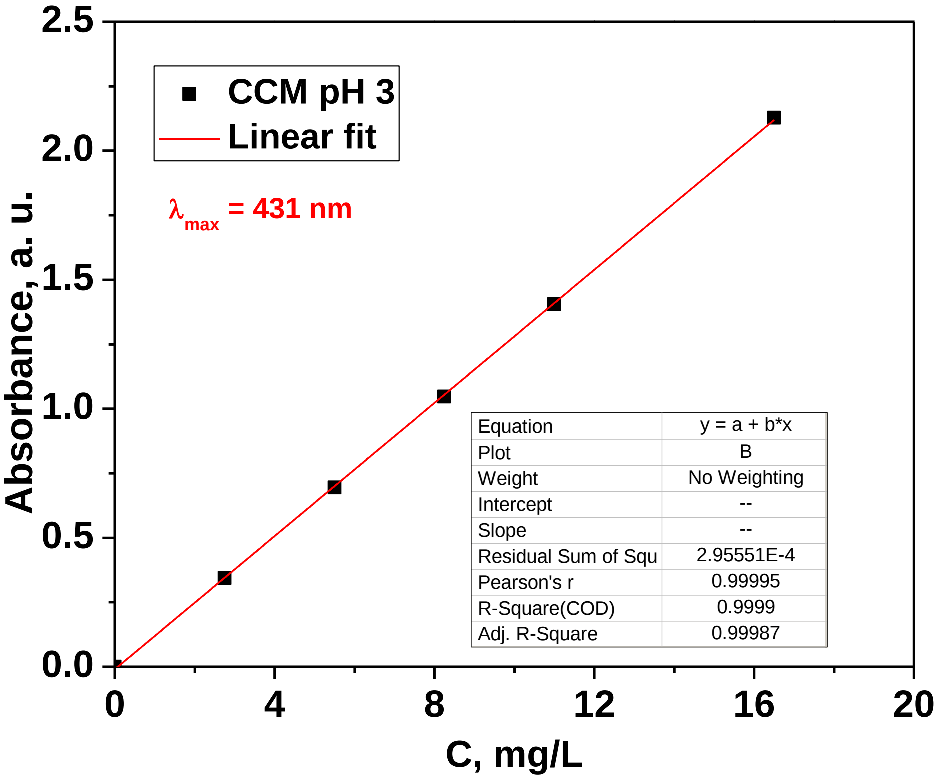

Figure S3. Calibration curve of CCM in ethanol: pH 3 aqueous solution, 50:50 (v/v).

Table S2. The mathematical formula of kinetic models.

**Kinetic model**	**Mathematical formula**
First-order	$M_t = M_0 . \mathit{exp}^{-k_1 . t}$
Higuchi	$Mt = k_t^{1/2}$
Korsmeyer-Pepas	$M_t/M_{inf} = k_{KP} . t^n$
